# Development and validation of a Knowledge, Attitudes and Practices (KAP) questionnaire for healthcare professionals on environmental sustainability in healthcare in Southern Africa

**DOI:** 10.12688/f1000research.157487.1

**Published:** 2024-10-31

**Authors:** Helga Lister, Karien Mostert, Tanita Botha, Emma Field, Danté Knock, Natasha Mubi, Stefani Odendaal, Megan Rohde, Filip Maric

**Affiliations:** 1 Department of Occupational Therapy, University of Pretoria Faculty of Health Sciences, Pretoria, Gauteng, South Africa; 2 Department of Physiotherapy, University of Pretoria Faculty of Health Sciences, Pretoria, Gauteng, South Africa; 3 Department of Statistics, University of Pretoria Faculty of Natural and Agricultural Sciences, Pretoria, Gauteng, South Africa; 4 Departmet of Health and Care Sciences, UiT The Arctic University of Norway Faculty of Health Sciences, Tromsø, Troms, Norway

**Keywords:** Content validity, knowledge attitudes and practices, healthcare, environmental sustainability, Southern Africa, questionnaire development

## Abstract

**Background:**

Global environmental degradation is increasingly driving poor health outcomes worldwide. Healthcare systems and services are often not environmentally sustainable and compound the problem, while healthcare professionals are also recognised as key leaders in advancing sustainable healthcare. To adopt this leadership position, healthcare professionals’ knowledge, attitudes, and practices regarding environmental sustainability in healthcare must be established. This article reports the development and validation of a new instrument for this purpose that corresponds to the specificities of the Southern African context.

**Methods:**

Questionnaire development followed a seven-stage process. Information was obtained from a 2021 study titled ‘South African Healthcare Professionals’ Knowledge, Attitudes, and Practices Regarding Environmental Sustainability in Healthcare: A Mixed-Methods Study’ to develop the instrument. Information was also sourced from the literature regarding environmental sustainability and healthcare to generate the first questionnaire with 29 items. The following stages included two rounds of expert input, separated by a pilot study with the target population to receive feedback regarding the instrument’s structure, relevance, and length. Content validity was determined through statistical analysis.

**Results:**

Feedback was received from nine experts in stage two and 13 pilot-study participants in stage four and incorporated to improve the questionnaire. In stage six, the questionnaire was rated by seven experts. The content validity index of the questionnaire was calculated at two different stages, after which the indices were compared. Following a final edit, the questionnaire has 24 questionnaire items. The closing analysis calculated the scale content validity index average (S-CVI/Ave) of 0,922; this indicates that the final questionnaire has excellent content validity.

**Conclusion:**

A questionnaire that assesses the knowledge, attitudes and practices of healthcare professionals regarding environmental sustainability in Southern Africa has been developed and validated. This questionnaire can now be used for further studies in Southern Africa.

## Introduction

Global environmental degradation includes increasing global warming, freshwater shortages, loss of biodiversity, and exhaustion of natural resources, amongst others. The effects of global environmental degradation are becoming increasingly evident. ^1– 4^ Noncommunicable diseases account for 38 million deaths annually, and many of the risk factors of these are thought to have an environmental origin. ^5^ Despite substantial and alarming evidence that biodiversity loss and climate change are inextricably linked to poor health outcomes, globally, many people are unaware of these far-reaching implications. A 2018 literature review demonstrated that most healthcare professionals realise that global environmental degradation is occurring, and they are aware of its detrimental effects on people’s health; yet, many healthcare professionals also recognise that they have inadequate knowledge on the topic. ^6^

The World Health Organisation defines the healthcare sector as “all organisations, institutions and resources that are devoted to producing health actions”. ^7^ Health actions have the chief purpose of improving health and are rooted in the ethical principle of “do no harm”. ^8^ Ironically, many actions and practices in healthcare compound the effects of global environmental change and its associated health risks through their environmental footprint. ^9^ In 2016, healthcare professionals were identified as key leaders in combatting the effects of global environmental degradation. ^10^ Several ways in which healthcare professionals can take action have been highlighted, including raising awareness of environmental change as a universal health matter, advocating for mitigation strategies and sustainable solutions in the health sector and surrounding environments, and advocating for vulnerable groups who experience health inequity related to global environmental degradation. ^10, 11^

Global surveys have been conducted to determine the perspective of healthcare professionals regarding global environmental degradation as a health concern. ^6^ Survey participants understood that global environmental degradation is happening and recognised that humans are its main driver. Participants felt responsible for educating the public and policymakers about the problem but identified personal, professional and societal barriers as preventing them from doing so. ^10^ Other various factors preventing healthcare professionals from adopting sustainable practices including a lack of knowledge, time constraints, the perception that their efforts would not make a worthwhile difference, limited finances and inadequate infrastructure. ^1, 6, 10, 12, 13^ Limited or non-existent policies, subsequent lack of implementation, and conflicts between environmental policies and policies focusing on economic development also affect the attainability of environmental sustainability. ^14^

Notably, African countries are usually poorly represented in such surveys with South Africa often providing the largest representation among African countries. ^6^ Southern African countries are particularly susceptible to global environmental degradation as they have interconnected social and environmental vulnerabilities. These include, for example, severe environmental degradation, high levels of inequality and poverty, an abundance of weather-sensitive diseases, limited infrastructure and limited financial means to effectively prepare for extreme weather events. ^15– 17^ Southern Africa thus faces extensive and compounding challenges related to global environmental degradation and its health impacts and needs better representation in relevant studies.

To unlock and increase the potential of Southern African healthcare professionals contributing to the improvement of environmental sustainability, a greater understanding of the current knowledge, attitude and practice (KAP) of healthcare professionals regarding environmental sustainability in healthcare is imperative. KAP studies are particularly useful in unfamiliar areas of research and are frequently used in healthcare as they can serve to combine both qualitative and quantitative data. ^18– 20^ In our research, studying KAP focused on gaining insight into **Knowledge:** what healthcare professionals know about environmental sustainability, **Attitude:** healthcare professionals’ feelings and opinions about environmental sustainability and **Practice:** what healthcare professionals do regarding environmental sustainability in practice.

In 2021, a study titled ‘South African Healthcare Professionals’ Knowledge, Attitudes, and Practices Regarding Environmental Sustainability in Healthcare: A Mixed-Methods Study’ was conducted at the University of Pretoria in the Health Sciences Faculty. ^21^ The study participants included registered South African audiologists, occupational therapists, physiotherapists, and speech-language pathologists. A convergent mixed-methods design was utilised with data collection in parallel quantitative and qualitative phases. A questionnaire was used in the quantitative phase to obtain information regarding the KAP of environmental sustainability in healthcare, while focus group discussions using open-ended questions were employed in the qualitative phase, followed by deductive thematic analysis of the discussion transcripts. ^21^ Although this study provides useful insights into the topic, the context was limited to the nation of South Africa. ^21^ To make the instrument applicable to the broader Southern African context encompassing Namibia, Zimbabwe, Angola, Eswatini, Lesotho, Zambia, and Mozambique, it is necessary to develop and validate a new KAP questionnaire that can serve as an accurate measure of the KAP of healthcare professionals in this unique geographic region.

This article’s research built on the results of our previous study to develop and validate an instrument for assessing the KAP of healthcare professionals on environmental sustainability in healthcare in Southern Africa. This questionnaire can provide invaluable insight into knowledge gaps, current practices, and healthcare professionals’ attitudes and willingness to take environmental responsibility and action. The inclusion of barriers and education sections in the questionnaire will additionally assist in addressing challenges and implementing sustainable healthcare education to advance environmental sustainability in Southern Africa. ^6^

## Methods

### Study design and setting

The study made use of a descriptive research design to develop and validate a KAP questionnaire as an instrument to collect quantitative and qualitative data on the KAP of healthcare professionals regarding environmental sustainability in healthcare in Southern Africa.

### Stages of the study and development of the questionnaire

The development and validation of the KAP questionnaire consisted of a six-stage process:

• Stage one: A mixed-methods study from 2021, together with updated literature, served as a starting point to generate the items used in the initial design of the first version of the questionnaire.
• Stage two: An expert panel evaluated the questionnaire
• Stage three: The expert feedback was analysed, and changes were incorporated into the questionnaire accordingly.
• Stage four: The pilot-study participants, consisting of the target population, completed the questionnaire and provided feedback to further improve the questionnaire.
• Stage five: Similar to the expert feedback, the feedback from the pilot study was analysed, and changes were incorporated into the questionnaire accordingly.
• Stage six: A final expert panel evaluated the questionnaire.
• Stage seven: The item ratings from the experts were used to validate the overall questionnaire. Following this, item removal took place, and a final validation of the questionnaire was performed.

### Study population and sampling

While developing and validating the instrument, the researchers recruited experts to participate in stage two (n=9) and stage six (n=7) of the study. These numbers were recommended by multiple comparable studies as a requirement for the expert panel to establish content validity. ^22– 24^ Non-probability purposive- and snowball sampling were used in the selection to identify and select experts appropriately based on the study’s inclusion criteria. ^25, 26^ The experts were recruited through social networking, using LinkedIn or ResearchGate, where both were identified as professional or academic social media platforms. To be considered as an expert, participants had to demonstrate expertise in their respective fields where sufficient expertise was defined through one or more of the following: holding academic qualifications at master, doctoral or higher level; being a researcher in a relevant subject field; having relevant publications and/or clinical experience. Here, relevant fields included: KAP studies/instrument development/validation studies, environmental sustainability, the Southern African context and healthcare from either of the following professions: physiotherapy, occupational therapy, speech-language pathology, audiology, human nutrition and dietetics, or nursing. Unlike the mixed-methods study of 2021, ^21^ the profession of human nutrition and dietetics was included in this study as this is a prevalent profession in delivering healthcare.

In stage four, stratified and snowball sampling were used to select the pilot-study participants. ^27^ The pilot-study participants had to be healthcare professionals in Southern Africa, working in any speciality of their profession, including physiotherapy, occupational therapy, speech-language pathology, audiology, human nutrition and dietetics, and nursing; practising for at least six months or working in academia. Emphasis was placed on recruiting participants from all the above-mentioned professions as well as all Southern African countries were represented among the included pilot-study participants.

The research team initially aimed to source 18 healthcare professionals as pilot-study participants, specifically two from each Southern African country. After distributing 34 ‘request to participate’ emails and sending numerous ‘follow-up’ emails, the research team received responses from 21 healthcare professionals who were willing to participate. The pilot-study participants were requested to complete the questionnaire and provide feedback. Despite sending reminders via email, only 13 pilot-study participants followed through with this.

### Developing questionnaire and item clarity

During the initial expert evaluation (stage two) and the pilot study (stage four), participants were requested to evaluate the entire instrument in terms of layout, instructions, length and/or time taken to complete the questionnaire. The experts were also requested to indicate whether they used a cellphone/mobile phone or laptop/computer to complete the questionnaire such that the research team could identify problems in completing the questionnaire on different common devices. The pilot study participants were divided in half, whereby seven of the pilot-study participants were requested to complete the questionnaire on their laptop/computer and the remaining six were requested to complete the questionnaire on their cell phone/mobile phone. This was done to ensure the accessibility of the questionnaire through various devices. Additionally, the experts also had the opportunity to provide any additional input regarding the content and the format. The pilot study participants were requested to provide overall feedback on the clarity and formulation of the questions.

During stages two and six, the experts followed a four-point satisfactory scale in terms of how satisfactory they considered each questionnaire item to be in measuring the construct and/or purpose of the research study: 1) unsatisfactory; 2) somewhat satisfactory, 3) quite satisfactory and 4) highly satisfactory. Additionally, they were requested to provide input as to how each item could be improved or rephrased if they provided a rating of one to three. This feedback was, however, only incorporated during stage three of the study and not in stage six. This data is still available for further questionnaire development.

The questionnaire was uploaded onto Qualtrics, a web-based tool, for data collection that allows for the creation of questionnaires and the analysis of their results. ^28^ This provided a link sent to participants to enable them to complete the questionnaire.

To receive feedback from the participants in the different stages, the researchers used Microsoft Word and Google Forms. During stage two, the information was electronically completed in Microsoft Word and tabulated into a Microsoft Excel document in stage three to ensure that the data could be easily accessed and compared. During stages four and six, feedback was requested on Google Forms, which automatically allowed the results to be analysed in Microsoft Excel.

### Evaluating content validity

The pilot study participants provided feedback regarding the clarity and formulation of the questions and the overall content of the questionnaire. The questionnaire was rated by the experts in both stage two and stage six. The item content validity index (I-CVI) refers to the average expert ratings of each questionnaire item. I-CVI ranges from zero to one and expresses a portion of agreement on the relevancy of each item. ^29^ The scale content validity index (S-CVI) was calculated using the average S/CVI (S-CVI/Ave) method, where the sum of all I-CVI scores is divided by the total number of questionnaire items.

In stage six, the final questionnaire was returned to the experts for a second and final round of feedback. Unfortunately, only seven out of the nine experts were available. In stage seven, following the calculation of both I-CVI and S-CVI/Ave, five questionnaire items were removed as they were rated with an I-CVI of lower than 0,79. One questionnaire item which received a rating of 0,714 was retained following researcher discussions, due to its relevance. The remainder of the questionnaire items all received an I-CVI greater than 0.79 (> 0.79). The final S-SCI/Ave was greater than 0.9 and ensured excellent content validity. ^29^

### Data analysis

Data analysis occurred in stages three, five and six of the questionnaire development. In stage three, the first round of expert feedback relating to each questionnaire item and the questionnaire as a whole was carefully analysed and discussed by the research team before making changes. In stage five, a similar approach was used in incorporating the feedback from the pilot study. In stage six, the experts rated the questionnaire items according to a four-point satisfactory scale; these ratings were used to determine the questionnaire’s content validity.

## Results

**Figure 1.**
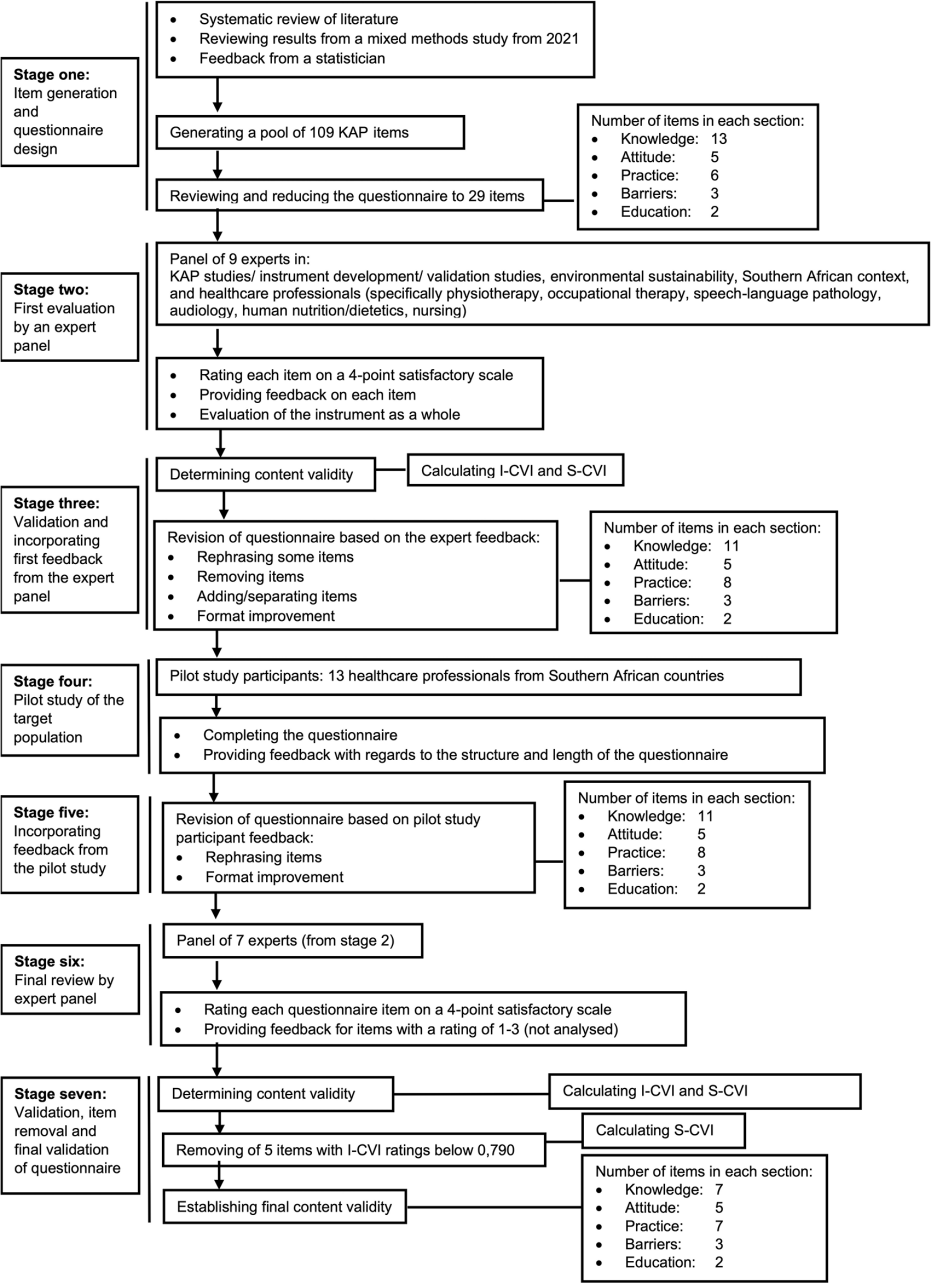
Line diagram of 7-stage process of KAP questionnaire development.

In this section, we present our findings according to each stage of the study. Figure 1 provides an overview of the seven stages of the questionnaire development and validation, together with a summary of each stage’s process.

### Stage one: Item generation and questionnaire design

In this stage, the research team evaluated and reviewed the results of the South African mixed-methods study. ^21^ The KAP questionnaire used in this study consisted of 14 close-ended and 11 open-ended questionnaire items. A total of 203 participants started the questionnaire, but only 100 participants completed the questionnaire, indicating a somewhat poor response rate. ^21^ In hindsight, this was considered as being a potential result of overly difficult knowledge-based questions and having too many open-ended questions. Next to sourcing additional and updated literature, the research team used the questionnaire items from the previous study as a basis for developing their pool of questionnaire items. The qualitative data was used to identify prevalent themes and categories. After this process, 109 potential questionnaire items were availed. To reduce this number and ensure relevance, the research team evaluated each questionnaire item individually. This iterative evaluation process was continued until agreement was reached on the final 29 questionnaire items, excluding the demographic section at the beginning of the questionnaire. Section one consisted of 13 knowledge-based questionnaire items, section two consisted of five attitudinal questionnaire items, and section three included six questionnaire items pertaining to environmental sustainability practices. The barrier and educational sections contained three and two questionnaire items, respectively.

### Stage two: Evaluation by an expert panel

The nine experts recruited fulfilled various combinations of the expert inclusion criteria. For instance, one of the experts demonstrated expertise in environmental sustainability, the Southern African context and as a healthcare professional. Of the nine experts, seven were healthcare professionals, two demonstrated expertise in KAP study/instrument development/validation studies, four demonstrated expertise in environmental sustainability, and two demonstrated expertise in the Southern African context. Regarding the demographics of the experts, six were female, and three were male, all ranging between the ages of 33 and 61.

The research team interpreted and analysed the expert ratings and the feedback that was provided. This process was methodical in that each questionnaire item, along with the expert feedback, was discussed individually. Before making the changes to the questionnaire items, the research team reached a consensus for amendments, removal or other measure to be taken for each questionnaire item.

### Stage three: Validation and incorporation of changes from the expert panel

During this stage, the I-CVI and S-CVI were calculated on the first draft of the questionnaire to determine a baseline for later comparison. As seen in Table 1, unfortunately, two questions were not evaluated by one of the experts. The S-CVI for section one was calculated at 0.749; for section two at 0.978 for section three at 0.924, section four at 0.963 and section five at 0.944. The S-CVI/ave for the whole questionnaire was 0.860.

**Table 1.** I-CVI and S-CVI calculations of questionnaire items after the first round of expert (n=9) feedback.

Question number	I-CVI (Item-Content Validity index)
Knowledge	
1.1 a	0.667
b	0.778
c	0.444
d	0.889
e	0.667
f	0.778
g	0.625
h	0.889
i	1
1.2 a	0.889
b	0.778
c	0.667
1.3	0.667
Average Scale CVI for knowledge section:	**0.749**
Attitudes	
2.1 a	0.889
b	1
c	1
d	1
e	1
Average Scale CVI for attitudes section:	**0.978**
Practices	
3.1	0.889
3.2	1
3.3	1
3.4	0.778
3.5	1
3.6	0.875
Average Scale CVI for practices section:	**0.924**
Barriers	
4.1	0.889
4.2	1
4.3	1
Average Scale CVI for barriers section:	**0.963**
Education	
5.1	0.889
5.2	1
Average Scale CVI for education section:	**0.944**
Average Scale CVI for the whole questionnaire *:	**0.86**

Note: 1.1g was only rated by 8 experts, not 9.

* Sum of all questions’ I-CVI divided by the total amount of questions.

Following this, the feedback was analysed and then used to change the questionnaire. Regarding the formulation of the instructions, one expert suggested that the phrase *‘based on your own knowledge’* be added to the instructions of the true or false questionnaire items in the knowledge section so that the participants understand that they are not expected to research their responses from a reference source, but to rather use their current knowledge.

Multiple experts noted that questionnaire items 1.1b, 1.1c, 1.1f and 2.1a contained too many theoretical constructs or different terminology, which may conflict with each other and therefore argued that this could confuse participants regarding what aspect of the questionnaire item they should refer to in answering it. For example, questionnaire item 1.1b originally read: *‘The impacts and risks of climate change on mental health are rapidly accelerating’.* The expert argued that ‘impacts and risks (are) not necessarily the same thing’. In response to this, the research team confined the questionnaire items listed above to one construct as far as possible, with for this question, the final item reading *‘Climate change has an impact on mental health’.*

One expert indicated that it could not be assumed that all participants completing the questionnaire believe that climate change has man-made origins with catastrophic effects. At the same time, participants can have certain beliefs whilst still being advocates of environmental sustainability. Therefore, the research team added an option to select *‘non-applicable’* in response to certain questionnaire items. Experts suggested that the option of *‘other’* be included for the questionnaire item *‘Climate change has a direct negative impact on human health, through which of the following?’* because some experts recognised additional negative human health impacts of climate change. Another expert noticed that two questionnaire items in the practice section, namely Q3.2 *‘Regardless of whether your place of work has a policy or not, which of the following strategies to improve environmental sustainability in healthcare have been implemented at your place of work?’* and Q3.5 *‘Which of the following environmentally sustainable measures for your place of work would you support?’* each listed different answering options for selection. The expert suggested that the same list of options be provided for both questionnaire items as this would create more flow. Therefore, the options were combined and edited so that they were applicable to both questionnaire items.

For the questionnaire item *‘What would be your preferred method of being educated on this topic as a practising professional?’* it was suggested to provide the option of *‘Continuous professional development (CPD) activities’* as this could be a means of gaining knowledge on the topic.

The expert panel further suggested that certain questionnaire items should be rephrased for greater clarity. For instance, it was suggested that the statement *‘environmental sustainability practices should be incorporated into healthcare’* was not specific enough, so the researchers amended this questionnaire item by specifying *‘into healthcare services’.* It was also suggested by multiple experts that *‘in your practice’* should be changed to *‘place of work’* to avoid ambiguity of the term ‘practice’, which is often related to ‘private practice’ and thus not applicable for those working in academia or a hospital. Some experts also suggested that a section should be created at the beginning of the questionnaire where definitions for key terms such as climate change and environmental sustainability are provided. The research team decided against this as it would indirectly disclose the answers for the knowledge section in advance and, consequently, participants’ knowledge would be inaccurately represented.

An expert also noticed that one could not backtrack between the questionnaire items. This expert suggested that for easier navigation and to allow participants to return to previous questionnaire items to provide the option to change their answers, backtracking should be allowed. The research team agreed with this suggestion and activated the backtracking function. A different expert recommended using larger text boxes where participants are allowed to type, such as in the case of selecting the option of ‘other’ for open-ended additions. The questionnaire was adapted accordingly. Although more than one expert proposed the use of Likert scales in more than just the attitudinal section, the research team decided against this recommendation in favour of the aim to derive rich information from participants, which would be limited by using a Likert scale. Minor spelling, numbering and punctuation errors were also highlighted and amended.

Several experts also suggested that the questionnaire items be more specific to ensure that participants understand what is being asked. Therefore, additional information was provided in brackets after certain questionnaire items and/or options. As requested, all acronyms were discarded and replaced by full terms.

Finally, all overall feedback was discussed and used to adapt the instrument and make necessary changes to individual questionnaire items. After the changes were incorporated, the questionnaire consisted of 11 knowledge, five attitudes, eight practice, three barriers and two education questionnaire items with a total of 29 questionnaire items.

### Stage four: Pilot study of the target population

Literature pertaining to the design and conduction of KAP questionnaires indicates that a small number of pilot-study participants (five to ten) are recommended as volunteers from the target population. ^26^ In this study, thirteen pilot-study participants provided feedback. They consisted of two participants from Zimbabwe and Lesotho, respectively; three participants from South Africa and Namibia, respectively; and one participant from Angola, Eswatini (Swaziland) and Mozambique, respectively. The pilot-study participants consisted of healthcare professionals in various fields, namely five nurses, four occupational therapists, one physiotherapist, one speech-language pathologist, one audiologist and one professional from human nutrition and dietetics. Six males and seven females between the ages of 24 and 66 participated in the study.

### Stage five: Incorporating feedback from the pilot study

In the feedback, all of the pilot-study participants stated that the questionnaire was easy to understand and clearly formulated and that they understood what was expected of them when answering the questionnaire items. Of the 13 pilot-study participants, 11 indicated that it did not take them longer than the approximate time stated, that being 15 to 20 minutes, to complete the questionnaire. Therefore, no changes were made to reduce the length of the questionnaire. Four pilot-study participants raised concerns with regard to the terminology used and suggested that simpler words be used to ensure understanding for those less proficient in the English language. Therefore, the research team examined the entire questionnaire and made necessary adjustments to ensure comprehension.

At this stage, the research team realised that participants from countries that do not have English as an official language, such as Angola and Mozambique, which both have Portuguese as their official language, would have difficulty understanding the questionnaire. Therefore, questions were included in the demographic section, instructing the participants to indicate their first (home) language as well as to rate their proficiency in English according to the provided options of poor, fair, good or excellent. No changes were made to the number of questionnaire items in this stage.

### Stage six: Final review by the expert panel

Final validation and review of the questionnaire was performed by the expert panel. Unfortunately, two experts from the first round of feedback were unavailable for the final round of feedback due to other commitments. Therefore, the final expert panel consisted of seven experts (n=7).

### Stage seven: Validation, item removal and final validation of the questionnaire

A statistician used the final expert ratings to calculate the Item-Content Validity Index (I-CVI) and the Scale-Content Validity Index (S-CVI). After the final evaluation by the expert panel, four questionnaire items from the knowledge section and one from the practice section, with ratings below 0.79, were removed from the questionnaire. An additional questionnaire item from the educational section also received a rating below 0.79. This questionnaire item required participants to select all methods that they consider applicable: ‘What would be your preferred method of being educated on this topic as a practising professional?’ The research team analysed this questionnaire item, and decided to retain it based on the shared conclusion that it would provide important information which could be used for intervention strategies in future studies.

As illustrated in Table two below, after calculating the S-CVI/Ave, the developed questionnaire has reached excellent content validity with a rating of 0.922. The S-CVI/Ave for each section was also calculated; the only section with a rating lower than 0.900 was the educational section with a rating of 0.857.

Notably, in the final expert feedback, some questionnaire items received lower S-CVI ratings than in the first round of feedback. Despite this, it was determined by comparing the S-CVI/Ave ratings from the first round of expert feedback ( Table 1) to those from the final round of expert feedback ( Table 2) that the validity of the questionnaire improved; with an initial S-CVI rating of 0.86 and a final rating of 0.922.

**Table 2.** I-CVI calculations of the questionnaire items by the expert panel (n=7).

Question number	I-CVI (Item-Content Validity index)
Knowledge	
1.1 a	1
b	1
c	0.71
d	0.857
e	0.857
f	1
1.2	1
Average Scale CVI for knowledge section:	**0.918**
Attitudes	
2.1 a	0.857
b	0.857
c	0.857
d	1
e	1
Average Scale CVI for attitudes section:	**0.914**
Practices	
3.1	0.857
3.2	0.857
3.3	1
3.4	1
3.5	1
3.6	1
3.7	1
Average Scale CVI for practices section:	**0.959**
Barriers	
4.1	0.857
4.2	0.857
4.3	1
Average Scale CVI for barriers section:	**0.905**
Education	
5.1	1
5.2	0.714
Average Scale CVI for education section:	**0.857**
Average Scale CVI for the whole questionnaire *:	**0.922**

Note: Questions for which less than 6 experts allocated a rating of 3 or 4 were removed, except for question 5.2 (which only 5 experts rated as 3 or 4). Due to its relevance, this question remained in the questionnaire following researcher consultations.

* Sum of all questions’ I-CVI divided by the total amount of questions.

## Discussion

There is limited literature regarding the KAP of healthcare professionals relating to the health impacts of climate change and environmental sustainability in healthcare, and healthcare professionals from the African continent are routinely not included in relevant studies. ^6, 21^ In the present study, a 24-item KAP questionnaire regarding environmental sustainability was developed and validated. The S-CVI/avg for the final questionnaire was calculated to be 0,922. An S-CVI of 0,800 is recommended as a minimum rating for a new instrument, and therefore, this study’s questionnaire exceeds the minimum S-CVI standard. ^29^

The research team could not draw comparisons from other studies because there is limited literature regarding the development and validation of KAP questionnaires pertaining to this topic. A methodologically similar study focussed on developing a questionnaire investigating the KAP among student tuberculosis patients in China. In this study, the questionnaire was distributed to an expert panel consisting of 12 experts who were expected to rate the questionnaire items on a five-point scale (where 0 = very unimportant and 4 = very important). Additionally, a two-round expert consultation was performed and CVI was used as a measure for content validity. This study also met the criteria for a content-valid questionnaire (0.962). ^30^ The similarities between the methodology employed in this and our study, particularly the use of an expert panel and two rounds of expert feedback, were deemed meaningful in that they demonstrated the successful use of the methodological approach.

The developed questionnaire positively contributes to the area of study as it addresses the recognised gap by availing a validated instrument which measures the KAP of healthcare professionals on environmental sustainability in healthcare in Southern Africa. By way of further contribution, the research team included questionnaire items that are not usually indicated in KAP studies, such as those in the barriers and education sections. This is pertinent because identifying and reducing the barriers that healthcare professionals face has the potential to increase the likelihood of implementation of practices advancing environmental sustainability. ^6^ In addition, data regarding healthcare professionals’ preferred methods on being educated on the topic can be obtained and inform future studies on and implementation of sustainable healthcare education.

## Conclusion

An instrument to assess the KAP of healthcare professionals in environmental sustainability in Southern Africa was developed and validated in this study. According to expert feedback and statistical calculations, it can be concluded that the questionnaire has an excellent content validity of 0.922 S-CVI/Ave. The questionnaire can therefore be considered as a valid instrument in measuring the KAP of healthcare professionals in environmental sustainability in Southern Africa. The data obtained through questionnaire employment may additionally serve to indicate barriers that prevent healthcare professionals from implementing sustainable practices and their views on sustainable healthcare education. Data collected from this questionnaire can be interpreted and used to develop appropriate and context-specific strategies to adopt more environmentally sustainable healthcare practices.

### Limitations

Despite extensive recruitment, the research team could not obtain any pilot study participants for Botswana and Zambia. Therefore, the questionnaire is not validated for these two Southern African countries. Additionally, two of the nine first round of experts (stage 2) were unavailable to assist in completing the validation of the questionnaire during the second round of expert feedback (stage 6). The research team also considered that some of the pilot-study participants did not have English as their first language and that the comprehension and interpretation of answers could have been misconstrued due to this language barrier.

### Recommendations

It is recommended that the questionnaire be validated in other regions across the world, commencing with the remainder of the countries in Sub-Saharan Africa, to determine its applicability across a wider geographical area. Together with this, it will be important to translate the questionnaire into the respective languages of each target population.

It is hoped that the questionnaire will be used by researchers from the respective countries in Southern Africa to determine a baseline of information from which various responses aimed at improving environmental sustainability in healthcare can be developed. Additionally, the employment of this can be a means of developing partnerships across Southern African countries - to share ideas, experiences, knowledge, and policies and implement changes.

## Ethical considerations

The study was conducted in accordance with the Declaration of Helsinki, and the research ethics committee of the University of Pretoria, Faculty of Health Sciences, granted permission to conduct the study (698/2021) on the 7th of February 2022. Informed consent was signed by all of the experts, while pilot-study participants clicked consent on the qualtrics form before proceeding with the questionnaire. Before doing so, both experts and participants had been provided information about the purposes and processes of the study and informed that their personal data would be anonymised. Anonymised data was used in the data analysis and interpretation of the findings. Ethical considerations were adhered to, including upholding confidentiality and anonymity.

## Consent

Written informed consent for publication of the participants/patients’ details and/or their images was obtained from the participants/patients/parents/guardians/relatives of the participant/patient.

## Data Availability

Figshare: First Round Expert Feedback, Ratings and Question Feedback, 10.6084/m9.figshare.21220283.v2. This project contains the following raw underlying data:
• First round expert feedback, ratings and question feedback (this dataset contains the ratings given by 9 experts for every questionnaire item as well as feedback on how each questionnaire item can be improved.) ^31^ Figshare: First Round Expert Feedback: General Feedback, 10.6084/m9.figshare.21220697.v2. This project contains the following raw underlying data:
• First round expert feedback: General feedback (this dataset contains general information provided by the experts about the layout, time taken to complete the questionnaire and additional feedback about the questionnaire.) ^32^ Figshare: First Round I-CVI and Average S-CVI, 10.6084/m9.figshare.21224900.v2. This project contains the following raw underlying data:
• First round I-CVI and average S-CVI (this document shows the item content validity index (I-CVI), as well as the average scale content validity index (S-CVI) calculated after the first round of expert feedback.) ^33^ Figshare: Pilot Study Participant Feedback, 10.6084/m9.figshare.21224918.v2. This project contains the following raw underlying data:
• Pilot study participant feedback (this dataset provides feedback from the pilot-study participants regarding language, clarity of questions, length of the questionnaire and user-friendliness.) ^34^ Figshare: Final Round Expert Feedback and Ratings, 10.6084/m9.figshare.21224954.v2. This project contains the following raw underlying data:
• Final round expert feedback and ratings (this dataset provides the final ratings given by the experts for the improved questionnaire items as well as additional feedback on how the questionnaire can be improved further.) ^35^ Figshare: Final Round Average I-CVI and S-CVI Calculation, 10.6084/m9.figshare.21224975.v2. This project contains the following raw underlying data:
• Final round average I-CVI and S-CVI calculation (the item content validity index (I-CVI) of the questionnaire items included in the final questionnaire as well as the average scale content validity index (S-CVI/Ave) of the final questionnaire.) ^36^ Data is available under the terms of the Creative Commons Zero “No rights reserved” data waiver (CC0). Final questionnaire on the knowledge, attitudes and practices of healthcare professionals on environmental sustainability in Southern Africa, 10.6084/m9.figshare.21224993.v2 This project contains the following raw underlying data:
• Final questionnaire on the knowledge, attitudes and practices of healthcare professionals on environmental sustainability in Southern Africa (the final questionnaire of 24 questionnaire items with excellent content validity.) ^37^ Data is available under the terms of the Creative Commons Zero “No rights reserved” data waiver (CC0).

## References

[1] Anabaraonye B Ewa BO Wala K : The Impacts of Climate Change on Nigeria’s Health Sector and Innovative Solutions for Environmental Sustainability. 2020;2:1–7.

[2] McMichael AJ Friel S Nyong A : Global environmental change and health: impacts, inequalities, and the health sector. *BMJ.* 2008 Jan 26;336(7637):191–194. 10.1136/bmj.39392.473727.AD 18219041 PMC2214484

[3] Romanello M Di Napoli C Drummond P : The 2022 report of the Lancet Countdown on health and climate change: health at the mercy of fossil fuels. *Lancet.* 2022 Nov 5;400(10363):1619–1654. 10.1016/S0140-6736(22)01540-9 36306815 PMC7616806

[4] World Health Organisation: *2021 WHO health and climate change global survey report.* Geneva: World Health Organization;2021.

[5] Frumkin H Haines A : Global Environmental Change and Noncommunicable Disease Risks. *Annu. Rev. Public Health.* 2019 Apr 1;40(1):261–282. 10.1146/annurev-publhealth-040218-043706 30633714

[6] Kotcher J Maibach E Miller J : Views of health professionals on climate change and health: a multinational survey study. *Lancet Planet Health.* 2021 May;5(5):e316–e323. 10.1016/S2542-5196(21)00053-X 33838130 PMC8099728

[7] Karliner J Slotterback S Boyd R : Health care’s climate footprint: the health sector contribution and opportunities for action. *Eur. J. Pub. Health.* 2020;30(Supplement_5). 10.1093/eurpub/ckaa165.843

[8] Rowan NJ Laffey JG : Unlocking the surge in demand for personal and protective equipment (PPE) and improvised face coverings arising from coronavirus disease (COVID-19) pandemic – Implications for efficacy, re-use and sustainable waste management. *Sci. Total Environ.* 2021 Jan;752:142259. 10.1016/j.scitotenv.2020.142259 33207488 PMC7481258

[9] Lenzen M Malik A Li M : The environmental footprint of health care: a global assessment. *Lancet Planet Health.* 2020 Jul;4(7):e271–e279. 10.1016/S2542-5196(20)30121-2 32681898

[10] Yang L Liu C Hess J : Health professionals in a changing climate: protocol for a scoping review. *BMJ Open.* 2019 Feb 1;9(2):e024451. 10.1136/bmjopen-2018-024451 30798312 PMC6398618

[11] World Health Organisation: *COP26 special report on climate change and health: the health argument for climate action.* Geneva: World Health Organization;2021.

[12] Hubbert B Ahmed M Kotcher J : Recruiting health professionals as sustainability advocates. *Lancet Planet Health.* 2020 Oct;4(10):e445–e446. 10.1016/S2542-5196(20)30225-4 33038317

[13] Ahmad S Eckelman MJ Sherman J : Environmental Impacts of the U.S. Health Care System and Effects on Public Health. *PLoS One.* 2016 Jun 9;11(6):e0157014. 10.1371/journal.pone.0157014 27280706 PMC4900601

[14] Howes M Wortley L Potts R : Environmental Sustainability: A Case of Policy Implementation Failure? 2017;18.

[15] Trewin B Adam JP Beltran J : State of the Climate in Africa. 2019.

[16] Ziervogel G New M Archer van Garderen E : Climate change impacts and adaptation in South Africa: Climate change impacts in South Africa. *Wiley Interdiscip. Rev. Clim. Chang.* 2014 Sep;5(5):605–620. 10.1002/wcc.295

[17] Meadows ME : Global Change and Southern Africa. *Geogr. Res.* 2006 Jun;44(2):135–145. 10.1111/j.1745-5871.2006.00375.x

[18] Andrade C Menon V Ameen S : Designing and Conducting Knowledge, Attitude, and Practice Surveys in Psychiatry: Practical Guidance. *Indian J. Psychol. Med.* 2020 Sep;42(5):478–481. 10.1177/0253717620946111 33414597 PMC7750837

[19] Torres-Vallejo Y Ruiz-Galeano CA Bonilla-Escobar FJ : Recommendations for Future Articles on Knowledge, Attitudes and Practices in IJMS. *Int. J. Med. Stud.* 2013 Dec 31;1(3):135–136. 10.5195/ijms.2013.227

[20] Muleme J Kankya C Ssempebwa JC : A Framework for Integrating Qualitative and Quantitative Data in Knowledge, Attitude, and Practice Studies: A Case Study of Pesticide Usage in Eastern Uganda. *Front. Public Health.* 2017 Dec 8;5:15. 10.3389/fpubh.2017.00318 29276703 PMC5727069

[21] Lister HE Mostert K Botha T : South African Healthcare Professionals’ Knowledge, Attitudes, and Practices Regarding Environmental Sustainability in Healthcare: A Mixed-Methods Study. *Int. J. Environ. Res. Public Health.* 2022 Aug 16;19(16):10121. 10.3390/ijerph191610121 36011760 PMC9408692

[22] Mallah N Rodriguez-cano R Figueiras : Design, reliability and construct validity of a Knowledge, Attitude and Practice questionnaire on personal use of antibiotics in Spain. *Sci. Rep.* 2020;10:20668. 10.1038/s41598-020-77769-6 33244041 PMC7693171

[23] Andrade C Menon V Ameen S : Designing and Conducting Knowledge, Attitude, and Practice Surveys in Psychiatry: Practical Guidance. *Indian J. Psychol. Med.* 2020;42(5):4. 33414597 10.1177/0253717620946111 PMC7750837

[24] Saefi M Fauzi A Kristiana E : Validating of Knowledge, Attitudes, and Practices Questionnaire for Prevention of COVID-19 infections among Undergraduate Students: A RASCH and Factor Analysis. *Eurasia J. Math., Sci Tech. Ed.* 2020;16(12):em1926. 10.29333/ejmste/9352

[25] Etikan I Musa S Alkassim R : Comparison of Convenience Sampling and Purposive Sampling. *Am. J. Theor. Appl. Stat.* 2016;5(1):1. 10.11648/j.ajtas.20160501.11

[26] Naderifar M Goli H Ghaljaie F : Snowball Sampling: A Purposeful Method of Sampling in Qualitative Research. Strides Dev. *Med. Educ.* 2017 Sep 30;14(3). 10.5812/sdme.67670 Reference Source

[27] Acharya AS Prakash A Saxena P : Sampling: why and how of it? *Indian J. Med. Spec.* 2013 Jul 7;4(4):330–333. 10.7713/ijms.2013.0032

[28] Cushman JE Faulkner R Fusso-Rollins M : Resource Review—Using Qualtrics Core XM for Surveying Youth. 2021.

[29] Rodrigues IB Adachi JD Beattie KA : Development and validation of a new tool to measure the facilitators, barriers and preferences to exercise in people with osteoporosis. *BMC Musculoskelet. Disord.* 2017 Dec;18(1):540. 10.1186/s12891-017-1914-5 29258503 PMC5738121

[30] Fan Y Jiang H Li Y : Development and psychometric testing of the Knowledge, Attitudes and Practices (KAP) questionnaire among student Tuberculosis (TB) Patients (STBP-KAPQ) in China. *BMC Infect. Dis.* 2018;18:213. 10.1186/s12879-018-3122-9 29739363 PMC5941627

[31] Lister H Mostert K Botha T : First round expert feedback, ratings and question feedback. *figshare.* 2022 [cited 2024 Oct 3]. Reference Source

[32] Lister H Mostert K Botha T : First round expert feedback: General feedback. *figshare.* 2022 [cited 2024 Oct 3]. Reference Source

[33] Lister H Mostert K Botha T : First round I-CVI and average S-CVI. *figshare.* 2022 [cited 2024 Oct 3]. Reference Source

[34] Lister H Mostert K Botha T : Pilot study participant feedback. *figshare.* 2022 [cited 2024 Oct 3]. Reference Source

[35] Lister H Mostert K Botha T : Final round expert feedback and ratings. *figshare.* 2022 [cited 2024 Oct 3]. Reference Source

[36] Lister H Mostert K Botha T : Final round average I-CVI and S-CVI calculation. *figshare.* 2022 [cited 2024 Oct 3]. Reference Source

[37] Lister H Mostert K Botha T : Final questionnaire on the knowledge, attitudes and practices of healthcare professionals on environmental sustainability in Southern Africa. *figshare.* 2022 [cited 2024 Oct 3]. Reference Source 10.12688/f1000research.157487.2 PMC11885900 40060749

