# Supplementary material for: Development and validation of a Knowledge, Attitudes and Practices (KAP) questionnaire for healthcare professionals on environmental sustainability in healthcare in Southern Africa
Source: F1000Res. 2025 Mar 26;13:1308. Originally published 2024 Oct 31. [Version 2] doi: 10.12688/f1000research.157487.2 (PMC11885900; doi:10.12688/f1000research.157487.2)
Supplement: Peer review history

# Reviewer report: Reviewer response for version 2

**DOI:** 10.5256/f1000research.179258.r373261

**Published:** 2025-03-27

**Authors:** Paolo Lauriola

**Affiliations:** 1 International Society Doctors for the Environment (ISDE), Rete Italiana Medici Sentinella per l'Ambiente (RIMSA), Arezzo, Italy

Thank you for your response. I am satisfied with the changes and agree the article.

Is the work clearly and accurately presented and does it cite the current literature?

Yes

If applicable, is the statistical analysis and its interpretation appropriate?

No

Are all the source data underlying the results available to ensure full reproducibility?

Yes

Is the study design appropriate and is the work technically sound?

Partly

Are the conclusions drawn adequately supported by the results?

Partly

Are sufficient details of methods and analysis provided to allow replication by others?

Yes

Reviewer Expertise:

Environmental Epidemiology, Public Health

I confirm that I have read this submission and believe that I have an appropriate level of expertise to confirm that it is of an acceptable scientific standard.

# Reviewer report: Reviewer response for version 1

**DOI:** 10.5256/f1000research.172940.r363888

**Published:** 2025-03-06

**Authors:** Jonathan Dixon

**Affiliations:** 1 School of Clinical Dentistry, University of Sheffield, Sheffield, UK

Thank you for the opportunity to review this interesting piece of research. The work is well presented and overall I am happy with the quality of the submission. A robust approach to questionnaire validation has been applied. My main suggestion is to provide further detail in the introduction and discussion sections that justify the need for this research. I appreciate the need but I am a researcher with expertise in the area – it may not be so obvious for other readers. Additionally, further clarity regarding the participants involved in the pilot would be beneficial (demographics etc)

 Congratulations to the authors – this was an interesting read.

Is the work clearly and accurately presented and does it cite the current literature?

Yes

If applicable, is the statistical analysis and its interpretation appropriate?

Yes

Are all the source data underlying the results available to ensure full reproducibility?

Yes

Is the study design appropriate and is the work technically sound?

Yes

Are the conclusions drawn adequately supported by the results?

Yes

Are sufficient details of methods and analysis provided to allow replication by others?

Yes

Reviewer Expertise:

Environmental sustainability in dentistry, Curriculum development

I confirm that I have read this submission and believe that I have an appropriate level of expertise to confirm that it is of an acceptable scientific standard.

# Response

**Published:** 2025-03-11

**Authors:** Filip Maric

**Affiliations:** Health and Care Sciences, UiT Norges arktiske universitet Det helsevitenskapelige fakultet, Tromsø, Troms, Norway

Thank you for your kind review. 

 Reviewer 2 also suggested that we provide further detail in the introduction and discussion sections that justify the need for this research. We have made resonant amendments in the introduction and discussion sections.

 We did not add additional demographic details because this is already provided under ‘Stage four: Pilot study of the target population’ in the results section.

# Reviewer report: Reviewer response for version 1

**DOI:** 10.5256/f1000research.172940.r363887

**Published:** 2025-02-26

**Authors:** Paolo Lauriola

**Affiliations:** 1 International Society Doctors for the Environment (ISDE), Rete Italiana Medici Sentinella per l'Ambiente (RIMSA), Arezzo, Italy

**General Comments**

 The intended use of the questionnaire remains unclear. It is crucial to explicitly state how the results will be utilized within local, national, and regional health organizations. Additionally, this article would benefit from a broader discussion on how the findings are expected to be implemented across these levels.

 Furthermore, the involvement of policymakers should be considered essential, as physicians play a critical role in raising public awareness regarding the importance of environmental sustainability. Engaging policymakers would enhance the questionnaire's impact, ensuring that its outcomes contribute effectively to knowledge enhancement, attitude shifts, and practical improvements in healthcare sustainability.

 By addressing these aspects, the questionnaire’s content could be more aligned with its ultimate objective of improving healthcare professionals' Knowledge, Attitudes, and Practices (KAP) regarding environmental sustainability.

**Specific Comments**

- **Keywords**: Please ensure that the keywords are distinct from those used in the title to enhance indexing and retrieval.
- **Introduction**: The article should reference the **Triple Planetary Crisis** (climate change, environmental pollution, and biodiversity loss) as defined by the United Nations Environment Programme (UNEP). A suitable citation would be: UNEP - Triple Planetary Crisis. (https://unfccc.int/news/what-is-the-triple-planetary-crisis)

Is the work clearly and accurately presented and does it cite the current literature?

Yes

If applicable, is the statistical analysis and its interpretation appropriate?

No

Are all the source data underlying the results available to ensure full reproducibility?

Yes

Is the study design appropriate and is the work technically sound?

Partly

Are the conclusions drawn adequately supported by the results?

Partly

Are sufficient details of methods and analysis provided to allow replication by others?

Yes

Reviewer Expertise:

Environmental Epidemiology, Public Health

I confirm that I have read this submission and believe that I have an appropriate level of expertise to confirm that it is of an acceptable scientific standard, however I have significant reservations, as outlined above.

# Response

**Published:** 2025-03-11

**Authors:** Filip Maric

**Affiliations:** Health and Care Sciences, UiT Norges arktiske universitet Det helsevitenskapelige fakultet, Tromsø, Troms, Norway

Thank you for your feedback.

 Hopefully, once published, researchers and policy makers interested in this field, will use the questionnaire in order to establish a baseline of information which can form a foundation for further work to enhance environmental sustainability in healthcare in Southern Africa, which is a in a particularly vulnerable area, to the effects of environmental degradation and climate change. A clarifying phrase and closing sentence has been added to the last paragraph in the introduction. Additionally, further information has been added to the final paragraph in the discussion section, explaining how the results of the questionnaire can be used. Specific strategies on how to implement the findings, is beyond the scope of this article. The second paragraph in the recommendations has been expanded upon, encouraging additional research and discussion amongst policy makers regarding how these findings can be implemented. Suggestions that have been made elsewhere have been referenced.

 Regarding the 'triple planetary crisis, the abstract opening sentence has been amended and the concept and reference have been added to the corresponding introductory paragraph.

# Reviewer report: Reviewer response for version 1

**DOI:** 10.5256/f1000research.172940.r355678

**Published:** 2025-01-28

**Authors:** Ebenezer Akore Yeboah

**Affiliations:** 1 Research Centre for Healthcare and Communities, Coventry University, Coventry, UK

Summary: Environmental sustainability in healthcare is quite a significant area needed to be explored to help protect and maintain the planet for all forms of life. Most commonly, research in this area is lacking in the global south especially Africa, hence it is significant to have seen and reviewed this paper on developing an instrument within African context. 

 My Review & Questions

 Well done to the authors. 

 1. Please check the third sentence in the third paragraph in the background for grammatical corrections.

 2. You indicated the medium of selection for the expert reviewers... Please what was the medium of participant selection for the pilot study?

 3. Under developing a questionnaire and item clarity in the methods section, you indicated below; "This feedback was, however, only incorporated during stage three of the study and not in stage six. This data is still available for further questionnaire development". Question; Were the feedback significant, if yes why not incorporated, if no why have you alluded to the need for that feedback in further development?

 4. At what stage of your process did you undertake a face validity of the instrument that is if it was performed, if no face validity why?

 5. In the questionnaire, 1a. climate change has a direct negative influence on human health

 1b. Climate change has an impact on mental health…

 Question: Is mental health not a subset of human health? Why have you emphasised mental health, or does 1a refer to human physical health? I would appreciate a response to this. 

 6. The word ‘ **Poverty stricken countries**’ is too harsh. A country's presence in the global south can make them vulnerable but they may not be poverty-stricken… Could you use a term like ‘low-income countries’ or 'countries with low adaptation plans' or global south countries or any other term which is friendly? 

 Thank you.

Is the work clearly and accurately presented and does it cite the current literature?

Yes

If applicable, is the statistical analysis and its interpretation appropriate?

I cannot comment. A qualified statistician is required.

Are all the source data underlying the results available to ensure full reproducibility?

Yes

Is the study design appropriate and is the work technically sound?

Yes

Are the conclusions drawn adequately supported by the results?

Yes

Are sufficient details of methods and analysis provided to allow replication by others?

Yes

Reviewer Expertise:

Nursing, Climate change, sustainability

I confirm that I have read this submission and believe that I have an appropriate level of expertise to confirm that it is of an acceptable scientific standard, however I have significant reservations, as outlined above.

# Response

**Published:** 2025-03-11

**Authors:** Filip Maric

**Affiliations:** Health and Care Sciences, UiT Norges arktiske universitet Det helsevitenskapelige fakultet, Tromsø, Troms, Norway

Thank you for your kind feedback. Here are our point-for-point responses (first part of the text are your points followed by our responses after hyphen series '---': 

1. Please check the third sentence in the third paragraph in the background for grammatical corrections.
  --- **Thank you. The sentence has been edited.**
2. You indicated the medium of selection for the expert reviewers... Please what was the medium of participant selection for the pilot study?
  --- **Thank you. The medium of selection has been added in the second sentence of the paragraph.**
3. Under developing a questionnaire and item clarity in the methods section, you indicated below; "This feedback was, however, only incorporated during stage three of the study and not in stage six. This data is still available for further questionnaire development". Question; Were the feedback significant, if yes why not incorporated, if no why have you alluded to the need for that feedback in further development?
**---  The feedback was not incorporated into the questionnaire for two reasons. Firstly, most of the suggestions were stylistic and grammatical in nature. Secondly, other suggestions were only provided by one or two experts, and thus not congruent across the experts. Since the final version was used to calculate the validity index, it was important that no further changes be made to the questionnaire. We decided to make the suggestions available for other researchers, so that these can be used if researchers from other regions want to validate the questionnaire for their region and wish to make further changes to it before the validation process. Explanatory sentences have been added to this section in the article.**
4. At what stage of your process did you undertake a face validity of the instrument that is if it was performed, if no face validity why?
**--- Thank you for highlighting this. In light of the thorough process undertaken to establish content validity, a detailed process of face validity was not conducted. However, the pilot study participants did provide positive “additional feedback” regarding the content of the questionnaire, for example indicating “the questions were in agreement with the study”, however since this was not formally addressed as face validity, it has not been specifically included in the article. This is thus a limitation and has been added under the limitation section in the article.**
5. In the questionnaire, 1a. climate change has a direct negative influence on human health. 1b. Climate change has an impact on mental health… Question: Is mental health not a subset of human health? Why have you emphasised mental health, or does 1a refer to human physical health? I would appreciate a response to this.
**--- Thank you. In the Southern African context, for the general population, when speaking of health, it is understood as physical health. Since mental health is still largely underrepresented and not recognised well, we therefore made a specific separate question to address it and ensure that the respondents think about mental health.**
6. The word ‘Poverty stricken countries’ is too harsh. A country's presence in the global south can make them vulnerable but they may not be poverty-stricken… Could you use a term like ‘low-income countries’ or 'countries with low adaptation plans' or global south countries or any other term which is friendly?
**--- Thank you. Whilst we agree with you regarding this point, the terminology for the questions were derived, amongst others, from the literature searches. There is a lot of literature referring to “poverty-stricken countries” being most vulnerable to the effects of climate change. Additionally, this highlights, the contrast of “wealthy” countries producing the majority of carbon emissions, whilst “poverty-stricken  countries” are more disadvantaged by the effects of these carbon emissions. Since the questionnaire has now been validated, we are unable to make additional changes to the questions.**

Kind regards,

 The authors
